# Strategic cyber intelligence with advanced analytics in Latin America: a perspective

**DOI:** 10.3389/fdata.2026.1733733

**Published:** 2026-05-01

**Authors:** Tamara Briones-Lascano, Vanessa Vergara-Lozano, Alex Miranda-Andrade

**Affiliations:** Facultad de Ciencias Agrarias, Universidad Agraria del Ecuador, Guayaquil, Ecuador

**Keywords:** advanced analytics, cyber intelligence, cybersecurity governance, digital resilience, Latin America

## Abstract

Digital transformation in Latin America has widened the attack surface, while exposing long-standing gaps in policy, capability, and data stewardship. From this perspective, we argue that the region can move from reactive cybersecurity to strategic cyber intelligence by embedding advanced analytics into an intelligence cycle that connects multisource data, governed models, and operational playbooks with clear accountability. We synthesize the demonstrated technical gains, diagnose implementation constraints, and outline a near-term agenda that includes a regional maturity index, comparative outcome studies, and decision research on explainability and bias. Thus, our position was practical. Analytics amplifies a good strategy only when governance, trustworthy data, and skilled teams are in place. This Perspective contributes a strategic analytical framework that links advanced analytics, governance, and decision-making to strengthen cyber intelligence and digital resilience in Latin America.

## Introduction

Cybersecurity in Latin America is no longer a specialist's concern. Incidents translate into measurable losses, service disruptions, and risk to rights that affect ministries, utilities, banks, and everyday users. Policy conversation has matured, yet outcomes remain uneven across countries and sectors. Our thesis is that strategic cyber-intelligence, supported by advanced analytics, provides the right organizing frame to anticipate threats, prioritize mitigation, and guide action. We use the term strategic to signal a system rather than a tool, a system where people, processes, and platforms align to produce timely, decision-ready intelligence.

We pursue the following three objectives. First, we synthesize advances in analytics that shorten the detection and response windows. Second, we grounded these advances in the region's institutional realities and offered a diagnostic that distinguishes exposure, capacity, and outcomes. Third, we propose directions for the next 3 years that can be adopted by security teams, regulators, and operators, without waiting for perfect conditions. Throughout, we take clear positions on what works and what does not, and connect technical promises with the constraints of institutions that must deliver essential services at scale.

## What analytics already do well

Recent applications of supervised and unsupervised learning, anomaly detection, and natural language processing have led to the earlier identification of lateral movement, credential abuse, and phishing campaigns that bypass legacy controls. In cloud and hybrid environments, these models improve visibility and accelerate triage, which are important for critical infrastructure and mission services (Lavayen Moran and Alvarado Sañudo, 2025; Parrales and Pinela, 2025). Practical value emerges when analytics is embedded in an intelligence cycle that normalizes data, separates signals from noise, generates testable hypotheses, and routes findings with explicit thresholds for action. Peer-reviewed research shows that machine learning-based intrusion detection and phishing classification can outperform signature-based approaches when deployed with stable data pipelines and continuous validation, although performance degrades in highly dynamic environments (Buczak and Guven, 2016; Sommer and Paxson, 2010). Table 1 summarizes the stages of the strategic cyber-intelligence cycle and clarifies the analytical purpose and typical outputs associated with each stage.

**Table 1:** Components of the strategic cyber-intelligence cycle and their analytical functions.

Cycle stage	Description	Analytical purpose	Typical outputs
Multisource data collection	Aggregation of heterogeneous cyber data from operational systems and external sources	Capture relevant signals across technical, organizational, and contextual domains	Logs, alerts, incident records, threat reports
Data normalization and feature engineering	Cleaning, standardization, enrichment, and alignment of data using shared taxonomies	Enable comparability, reproducibility, and reliable modeling	Structured datasets, engineered features
Advanced analytics	Application of statistical and machine learning methods to processed data	Identify patterns, anomalies, and trends relevant to cyber risk	Risk scores, classifications, anomaly flags
Intelligence products	Translation of analytical outputs into decision-relevant insights	Support prioritization and hypothesis generation	Alerts, risk assessments, threat profiles
Decision and action	Operational, managerial, or policy responses informed by intelligence	Allocate resources and implement mitigation measures	Containment actions, policy adjustments
Feedback and learning	Systematic review of outcomes and model performance	Improve analytical accuracy and governance over time	Model retraining, revised thresholds, updated playbooks

We take a position against viewing analytics as a standalone purchase that can be dropped into an unchanged organization. Advantages are perishable because offense and defense coevolve. Adversaries weaponize similar techniques to craft believable phishing at scale, obfuscate malware, and use probes for detecting blind spots. Therefore, we emphasize out-of-sample validation, drift monitoring, reproducible pipelines, and disciplined retraining as the minimum durability values. We also argue that model outputs must be paired with playbooks that specify who decides, within what time budget, and with which fallback, if confidence is low. Without coupling between analytics and decision responsibility, alerts become noise, and teams revert to manual heuristics that do not scale (Araya, 2023). This view is consistent with cyber threat intelligence scholarship, which emphasizes that analytical outputs generate value only when embedded within structured intelligence cycles linking collection, analysis, dissemination, and decision-making (Hutchins et al., 2011; Ruan, 2017).

Another lesson is the small design compound. The manner in which a team defines false positives and false negatives in phishing filters, the aggregation window for anomaly scores, or the choice of baselines for user behavior analytics can determine whether an operations center focuses on the five alerts that matter or drowns in a thousand. We recommend beginning with a narrow set of use cases that tie directly to outcomes, for example, reducing the mean time to detect by a defined percentage in a high-value business process, and then expanding once teams have learned how data, models, and playbooks interact in production.

## A diagnostic of Latin America: exposure, capacity, and outcomes

The exposure increases faster than the global baselines. Global risk assessments indicate that Latin America and the Caribbean face elevated cyber risk relative to their level of digital adoption, reflecting rapid digitization combined with uneven institutional and technical capacity (World Economic Forum, 2024). Mid-year assessments in 2025 reported that organizations in Latin America faced weekly attack volumes significantly above the global average, continuing an upward trend since 2024. These patterns indicate that adversaries view the region as an attractive and expanding target set, particularly where digital payments, identity systems, and cloud migrations have advanced faster than governance and workforce development (Check Point Research, 2025; Nelson, 2025).

These outcomes make the exposure more concrete. In 2022, Costa Rica declared a national emergency after ransomware operations disrupted dozens of government entities including the finance ministry and social security services. In 2023, a ransomware incident involving a major regional network provider impaired multiple Colombian government services. Large enterprises have also been affected, including Chile's Bank Estado in 2020 and Mexico's Pemex in 2019. These episodes revealed dependencies on shared providers, limited segmentation, and uneven incident reporting across jurisdictions. They also highlight the regional character of many incidents, as a compromise of a shared vendor can cascade across borders, even when national teams operate at different maturity levels (Burgess, 2022; Greig, 2023; Harán, 2022; Infobae, 2020; Stillman, 2019).

These cases illustrate not isolated events but recurrent structural vulnerabilities associated with shared service providers, limited segmentation, and uneven reporting practices, which amplify systemic risk at the regional level.

Capacity indicators help explain the persistent impact. According to the International Telecommunication Union Global Cybersecurity Index, countries in Latin America and the Caribbean exhibit uneven development across cybersecurity dimensions, with comparatively weaker performance in technical capacity, organizational measures, and workforce development than in legal and policy frameworks (Global Cybersecurity Index, 2021). The joint assessment by the Inter-American Development Bank and the Organization of American States found that as of 2020, only a subset of countries in Latin America and the Caribbean had approved national cybersecurity strategies, fewer had critical infrastructure protection plans, and national incident response teams were still consolidating. Similarly, the International Telecommunication Union's Global Cybersecurity Index documents uneven development across legal, technical, organizational, capacity-building, and cooperation pillars (Global Cybersecurity Index, 2021; Porrúa et al., 2025). National trajectories show progress, for example in Colombia and Panama, yet implementation remains heterogeneous and often dependent on short funding cycles and rotating leadership that interrupt continuity in programs and teams (Inter-American Commission on Human Rights, 2024; Ministerio de Educación Nacional, 2021; Ministerio de Tecnologías de la Información y las Comunicaciones, 2021).

We draw three implications from this diagnostic approach. First, the risk concentration is real. A handful of shared providers, identity hubs, and payment rails create a single point of failure. Second, the measurements were weak. Many countries cannot produce consistent time series for the mean time to detect incident recurrence or loss by sector, which limits policy learning. Third, the incentives are misaligned. Underreporting is rational when penalties are clear and the benefits are diffuse. Therefore, progress depends on targeted transparency policies, safe reporting channels, and metrics that reward meaningful reductions in risk rather than compliance with paperwork. These indicators suggest that elevated exposure interacts with constrained institutional capacity to generate disproportionately severe outcomes, rather than reflecting isolated or exceptional incidents.

## Techniques and what it takes to operationalize them

Advanced analytics offers a flexible toolkit for regional teams. Classification improves phishing and malware triage. Unsupervised methods surface low-prevalence anomalies in high-volume telemetry. Time-series models support capacity planning and incident trend analysis. Natural language processing helps parse advisories and threat reports, accelerating vulnerability prioritization. These capabilities are within reach of properly staffed teams, but three constraints determine whether they deliver value at scale in Latin America.

The first constraint is data availability and quality. Underreporting, inconsistent taxonomies, and a scarcity of labeled datasets complicate the detection of rare events and hinder transfer learning across sectors and countries. We advocate national and sectoral taxonomies that align with international standards and are adopted in procurement and supervision so that they become the default language of incident recording. The second constraint is the human capacity. Teams must translate regulatory and mission requirements into data design, reproducible pipelines, and governed models with clear privacy and explainability boundaries. We support curricular updates that emphasize data engineering for security, model governance, and domain knowledge and recommend residencies inside ministries, utilities, and financial market infrastructures where students and early career professionals can practice under supervision (Binario, 2025). The third constraint is organizational adoption. Cost concerns, regulatory uncertainty, and capability gaps delay deployment and widen the distance between academic evidence and production. We have seen that early wins in a few critical processes, openly measured and communicated, and unlock support for the next round of investment and hiring (Beer et al., 1990; Kotter, 2007).

We also address a build-vs.-buy question that often stalls. We are skeptical of total outsourcing for core detection and intelligence functions. Managed services can accelerate baselining, but strategic competence should remain in-house to preserve the context, accountability, and learning loops. A hybrid approach, in which external partners supply commodity detection and enrichment, while internal teams own use case selection, data models, and playbooks, offers a practical balance for resource-constrained organizations.

## Data stewardship, ethics, and rights

Analytics in security contexts requires foundational safeguards. Data protection, lawful bases for processing, proportionality, and model transparency are the conditions for legitimacy rather than optional add-ons. We recommend four regional deployment practices. Teams should conduct algorithmic impact assessments proportionate to risk; maintain auditable records of automated decisions; establish intersectoral oversight that connects regulators, incident responders, operators, prosecutors, and civil society; and sustain digital literacy programs that explain how model-assisted decisions are made. These measures align with regional legal trajectories and function as enablers of analytical capability rather than constraints on it (Araya, 2023). These concerns also align with the broader literature on AI governance, which highlights risks related to opacity, bias, and accountability when algorithmic systems are deployed in high-stakes socio-technical contexts (Floridi et al., 2018; Mittelstadt et al., 2016).

We also recognize the tension between privacy and effective detection. Our position is that careful data minimization and purpose limitations can coexist with useful analytics when teams invest in feature engineering that encodes patterns without exposing unnecessary personal identifiers. Differential privacy and synthetic data are not cured of all problems, yet they can expand access to training material for benchmarking and education when paired with governance that prevents reidentification and misuse.

## Building capabilities: people, process, and platforms

Technology does not move the needle if teams and processes are misaligned. We advocate multidisciplinary teams that combine security analysts, data engineers, applied scientists, and legal and ethics advisors who train in realistic scenarios. Processes should define intake, triage, escalation, and closure with service-level targets and feedback loops to improve features and labels. Platforms should support end-to-end handling, from collection and normalization to feature stores, model registries, and explainability tools instrumented with performance and drift monitoring.

We found that two practices were underused and had a high yield. The first is a postmortem model. After a significant miss or major false positive, teams should write a short, blameless narrative that traces data lineage, feature behavior, thresholds, and decision paths. The second is the red team exercise for analytics, in which adversarial testers intentionally craft traffic and artifacts to probe blind spots. Both practices convert isolated incidents into shared learning, and align engineers, analysts, and leadership around concrete improvements.

Education can speed this transition if curricula integrate data engineering, model governance, and sector knowledge and if residencies place students inside ministries, regulators, and utilities to build tacit expertise. We support the creation of applied learning laboratories where students, faculty, and practitioners work on real datasets under confidentiality agreements that respect law and ethics while delivering practical outcomes for host institutions.

## Policy, cooperation, and the role of measurement

Policy frameworks have advanced; however, common metrics remain scarce. We see value in the regional maturity index for cyber intelligence that blends governance quality, data stewardship, analytical depth, and outcome measures. Methodologically, the proposed regional cyber-intelligence maturity index is structured around four dimensions: governance, data stewardship, analytical capability, and outcomes. Governance refers to the existence and coordination of strategies, mandates, and oversight mechanisms; data stewardship captures data availability, standardization, and protection practices; analytical capability includes tooling, skills, and model governance; and outcomes focus on detection timeliness, containment effectiveness, and incident recurrence. Indicative data sources include national cybersecurity strategies, incident response disclosures, and international indices such as the ITU Global Cybersecurity Index. Validation would rely on cross-country benchmarking, expert review, and repeated measurement over time.

This instrument should be transparent, reproducible, and periodically published to incentivize improvements. We recommend that it include both capacity indicators and outcome indicators, including mean time to detect, mean time to contain, incident recurrence, loss estimates by sector, and the share of incidents that involve third-party or supply chain compromise.

Cooperation must become routine, rather than episodic. National teams, regulators, prosecutors, infrastructure operators, and universities should sustain joint exercises and information exchanges with clear objectives and success measures. International instruments and operational agreements reduce friction for cross-border investigations and lawful data sharing, which is essential in regions where many incidents have transnational footprint. We also encourage regulators to experiment with supervised sandboxes that allow critical infrastructure operators to validate analytics on production-like data without unacceptable risk and to use the evidence from those programs to refine guidance and procurement rules (Inter-American Commission on Human Rights, 2025; Vasciannie and Vasciannie, 2024).

## Directions for the next 3 years

We propose three lines of work with high short-term returns. The first is a regional cyber-intelligence maturity index that tracks governance, data, technical capability, and outcomes. The index should include a documented methodology, a permissible public data release, and a peer review process that invites challenges. The second is comparative outcome research that links the adoption of analytics with changes in the attack surface, containment times, and loss estimates by sector. These studies can move the debate from inputs to effects and help procurement and policy focus on interventions that work. The third is decision research on explainability, bias, and accountability, which studies how model outputs change operational and strategic choices without creating false confidence. We also support practical accelerants that include standardized models and data documentation, shared evaluation harnesses with representative regional datasets, and regulatory sandboxes supervised by competent authorities.

We have added a fourth, often overlooked, direction. Procurement reform can speed up adoption while raising quality. If requests for proposals require candidate solutions to disclose the model lineage, feature dependencies, retraining procedures, and expected data rights, buyers can compare options on substance rather than marketing. Contracts should align incentives by tying payment milestones to outcome improvements that matter to the mission, rather than to the number of dashboards delivered.

## Discussion

Evidence suggests that analytics can reduce detection and containment times and improve response prioritization. Whether these benefits materialize depends on institutional and data capacities that are distributed unevenly across Latin America. Where policy frameworks are consolidated, incident response is professionalized, data are stewarded, and analytical tools translate into measurable gains. Where norms are vague, talent is scarce, and coordination is weak, analytics tends to be underused. Therefore, we insist on reading cyber-intelligence as a system that integrates open and closed sources, descriptive and predictive analytics, vulnerability management, incident response, and a governance layer that defines limits, priorities, and responsibilities.

We take a clear stance on these controversies. Centralization vs. decentralization. We favor a federated approach, in which a national or sectoral hub defines taxonomies, data standards, and shared analytics, while agencies and firms retain operational discretion and context. Full centralization invites single points of failure and political interference, whereas full decentralization fragments learning and prevents economies of scale. Second, the transparency vs. security relationship is obscure. We support targeted transparency, including the publication of maturity metrics and sanitized postmortems for major incidents, because secrecy rarely protects the weaknesses that adversaries can infer from behavior. The third is automation vs. human judgment. We rejected both the extremes. Automation should handle volume and consistency, whereas human judgment should arbitrate ambiguous cases, review edge conditions, and decide when to override models based on mission impact and legal constraints.

Existing frameworks such as the NIST Cybersecurity Framework and MITRE ATT&CK provide essential guidance on controls, functions, and adversary techniques, while regional assessments produced by the Organization of American States and the Inter-American Development Bank focus on benchmarking national capacities. The approach proposed here does not seek to replace these instruments. Instead, it advances a strategic cyber-intelligence layer that connects advanced analytics with governance and decision accountability, emphasizing how analytical capabilities translate into institutional and policy outcomes over time.

We also address the risks that can reverse gains if ignored. Biased training data can tilt detection performance against certain user groups or network segments, producing blind spots where defenders expect them the least. Overfitting past incidents can create brittle models that degrade when attackers change their tactics. Unclear data rights can block retraining when contracts lapse or providers change terms. These risks are manageable with governance that requires a diversity of training sources, regular red teaming of models, and contracts that preserve access to features and labels generated by an organization's own operations.

Finally, we reflect on the cultural dimensions. Many teams inherit a compliance mindset that values exhaustive documentation of measurable outcomes. We promote a culture of disciplined experimentation in which hypotheses about detection performance and response times are tested with small, reversible changes and in which teams publish their internal findings to build trust across lines of responsibility. Culture is difficult to legislate, yet leadership can model it by celebrating learning from misses and by funding capacity that survives electoral or executive turnovers.

## Conclusion

Advanced analytics does not replace this strategy. They amplify it. With quality data and competent teams, analytics act as a force multiplier that helps anticipate, prioritize, and respond. In Latin America, the gaps and advances are real. The immediate challenge is the execution. Converting analytical promises into everyday resilience depends on the choices that the region can make. If institutions formalize cyber intelligence as a strategic function, invest in data and talent, and coordinate efforts with shared objectives and public metrics, then the region can move from reaction to anticipation, and from dispersed vulnerability to intelligent, collaborative defense.

## Data Availability

The original contributions presented in the study are included in the article/supplementary material, further inquiries can be directed to the corresponding author/s.
